# Iron Deficiency in Heart Failure and Effect of Dapagliflozin: Findings From DAPA-HF

**DOI:** 10.1161/CIRCULATIONAHA.122.060511

**Published:** 2022-08-16

**Authors:** Kieran F. Docherty, Paul Welsh, Subodh Verma, Rudolf A. De Boer, Eileen O’Meara, Olof Bengtsson, Lars Køber, Mikhail N. Kosiborod, Ann Hammarstedt, Anna Maria Langkilde, Daniel Lindholm, Dustin J. Little, Mikaela Sjöstrand, Felipe A. Martinez, Piotr Ponikowski, Marc S. Sabatine, David A. Morrow, Morten Schou, Scott D. Solomon, Naveed Sattar, Pardeep S. Jhund, John J.V. McMurray

**Affiliations:** British Heart Foundation Cardiovascular Research Centre, University of Glasgow, United Kingdom (K.F.D., P.W., N.S., P.S.J., J.J.V.M.).; Division of Cardiac Surgery, St Michael’s Hospital, University of Toronto, Canada (S.V.).; Department of Cardiology, University Medical Center and University of Groningen, The Netherlands (R.A.D.B.).; Montreal Heart Institute, Université de Montréal, Canada (E.O.).; AstraZeneca R&D, Gothenburg, Sweden (O.B., A.H., A.M.L., D.L., D.J.L., M. Sjöstrand).; Rigshospitalet Copenhagen University Hospital, Denmark (L.K.).; Saint Luke’s Mid America Heart Institute and University of Missouri-Kansas City (M.N.K.).; George Institute for Global Health, University of New South Wales, Sydney, Australia (M.N.K.).; National University of Cordoba, Argentina (F.A.M.).; Wroclaw Medical University, Poland (P.P.).; TIMI (Thrombolysis in Myocardial Infarction) Study Group, Cardiovascular Division, Brigham and Women’s Hospital, and Harvard Medical School, Boston, MA (M.S.S., D.A.M.).; Department of Cardiology, Gentofte University Hospital, Copenhagen, Denmark (M. Schou).; Cardiovascular Division, Brigham and Women’s Hospital, Boston, MA (S.D.S.).

**Keywords:** anemia, erythropoiesis, ferritin, heart failure, hepcidin, iron, sodium-glucose cotransporter 2 inhibitor, transferrin

## Abstract

Iron deficiency is common in heart failure and associated with worse outcomes. We examined the prevalence and consequences of iron deficiency in the DAPA-HF trial (Dapagliflozin and Prevention of Adverse-Outcomes in Heart Failure) and the effect of dapagliflozin on markers of iron metabolism. We also analyzed the effect of dapagliflozin on outcomes, according to iron status at baseline.

**Methods:**

Iron deficiency was defined as a ferritin level <100 ng/mL or a transferrin saturation <20% and a ferritin level 100 to 299 ng/mL. Additional biomarkers of iron metabolism, including soluble transferrin receptor, erythropoietin, and hepcidin were measured at baseline and 12 months after randomization. The primary outcome was a composite of worsening heart failure (hospitalization or urgent visit requiring intravenous therapy) or cardiovascular death.

**Results:**

Of the 4744 patients randomized in DAPA-HF, 3009 had ferritin and transferrin saturation measurements available at baseline, and 1314 of these participants (43.7%) were iron deficient. The rate of the primary outcome was higher in patients with iron deficiency (16.6 per 100 person-years) compared with those without (10.4 per 100 person-years; *P*<0.0001). The effect of dapagliflozin on the primary outcome was consistent in iron-deficient compared with iron-replete patients (hazard ratio, 0.74 [95% CI, 0.58–0.92] versus 0.81 [95% CI, 0.63–1.03]; *P*-interaction=0.59). Similar findings were observed for cardiovascular death, heart failure hospitalization, and all-cause mortality. Transferrin saturation, ferritin, and hepcidin were reduced and total iron-binding capacity and soluble transferrin receptor increased with dapagliflozin compared with placebo.

**Conclusions:**

Iron deficiency was common in DAPA-HF and associated with worse outcomes. Dapagliflozin appeared to increase iron use but improved outcomes, irrespective of iron status at baseline.

**Registration:**

URL: https://www.clinicaltrials.gov; Unique identifier: NCT03036124.

**Clinical Perspective**

**What Is New?**

- In the DAPA-HF trial (Dapagliflozin and Prevention of Adverse-Outcomes in Heart Failure), among patients with heart failure and reduced ejection fraction, the presence of iron deficiency at baseline was a risk factor for cardiovascular death and worsening heart failure events, including after adjustment for NT-proBNP (N-terminal pro-B-type natriuretic peptide) and high-sensitivity troponin T.
- In the DAPA-HF trial, the clinical benefits of dapagliflozin were consistent regardless of the presence or absence of iron deficiency at baseline.
- Dapagliflozin caused changes in biomarkers relating to iron metabolism consistent with an increase in iron mobilization and use, likely secondary to increased erythropoiesis.

**What Are the Clinical Implications?**

- The presence of iron deficiency should not be considered a barrier to the use of dapagliflozin in patients with heart failure and reduced ejection fraction.
- Regular monitoring of iron status should be performed in patients with heart failure and reduced ejection fraction, and guideline recommendations followed with regards to intravenous iron replacement when indicated.

Absolute and functional iron deficiency, the latter reflecting reduced iron mobilization and use, are common in patients with heart failure.^1–3^ The causes are thought to include reduced dietary intake, reduced absorption because of gut edema and use of drugs such as proton pump inhibitors, blood loss, and inflammation, which may inhibit both iron absorption and use.^1–3^ Some but not all patients with iron deficiency also have anemia.^1–3^ Patients with iron deficiency have worse symptoms and quality of life, greater functional impairment, and higher rates of hospital admission and death than those without iron deficiency, irrespective of anemia status.^1–4^ That iron deficiency is causally implicated in worse clinical status in patients with heart failure has been proven by the salutary effects of treatment with intravenous iron.^5–9^ Several randomized trials have shown improvement in symptoms, health-related quality of life, and exercise capacity with ferric carboxymaltose. Recently, the AFFIRM-AHF study (Study to Compare Ferric Carboxymaltose With Placebo in Patients With Acute Heart Failure and Iron Deficiency) demonstrated that intravenous iron also reduced the risk of readmission when given to patients hospitalized with worsening heart failure.^9^ Avoidance, detection, and treatment of iron deficiency are therefore recommended in contemporary heart failure guidelines.^10,11^

Iron deficiency may have implications for another new development in heart failure therapeutics. SGLT2 (sodium-glucose cotransporter 2) inhibitors have been shown to increase hematocrit and hemoglobin, and correct and prevent anemia in patients with heart failure.^12–14^ Although the increase in hematocrit was originally thought to reflect volume contraction caused by diuresis, it is now thought that SGLT2 inhibitors also stimulate erythropoiesis.^15–19^ Erythropoiesis uses iron and may be restricted by existing iron deficiency. This may be important if an increase in hematocrit contributes to the benefits of SGLT2 inhibitors, as has been suggested.^13,14^ Conversely, erythropoiesis may induce iron depletion, which is undesirable in heart failure, as discussed above, and some patients treated with an SGLT2 inhibitor might require concomitant iron replacement, analogous to the iron cotreatment required in patients with chronic kidney disease treated with an erythropoiesis-stimulating agent.^20,21^ The placebo-controlled DAPA-HF trial (Dapagliflozin and Prevention of Adverse-Outcomes in Heart Failure) provided a unique opportunity to examine the effect of an SGLT2 inhibitor on iron status in patients with heart failure and reduced ejection fraction (HFrEF) and the interaction between the effect of dapagliflozin on symptoms, quality of life, hospitalization and mortality, and iron status.^22,23^

## Methods

The efficacy and safety of dapagliflozin 10 mg once daily, compared with placebo, added to standard care was tested in patients with HFrEF in the prospective, randomized, double-blind, placebo-controlled DAPA-HF trial.^22,23^ Ethics committees at each participating site approved the protocol, and each patient gave written informed consent. The data that support the findings of this study are available from the corresponding author on reasonable request.

### Study Patients

Adults in New York Heart Association functional class II to IV, with a left ventricular ejection fraction (LVEF) ≤40% and an elevated NT-proBNP (N-terminal pro-B-type natriuretic peptide) level, optimally treated with pharmacological and device therapy, according to local guidelines, were eligible. The key exclusion criteria included the following: symptoms of hypotension or systolic blood pressure <95 mm Hg, estimated glomerular filtration rate (eGFR) <30 mL·min·1.73 m^2^, and type 1 diabetes. There was no exclusion related to iron, anemia, or hemoglobin/hematocrit. A full list of exclusion criteria is provided in the design article.^22^

### Measurement of Markers of Iron Metabolism and Other Biomarkers

Hematocrit was measured at baseline, as well as 14 days, 2 months and 4 months after randomization, and every 4 months thereafter. Hemoglobin was measured at baseline and at the end of the trial, but not routinely after randomization.

Biomarkers relating to iron metabolism were measured in a central laboratory (University of Glasgow) at baseline and 12 months after randomization. Not all countries participated in the DAPA-HF biomarker substudy; therefore, the results presented include only those with available measurements. Serum ferritin, unsaturated iron-binding capacity, iron, and soluble transferrin receptor (sTFR) were measured using an automated platform and the manufacturer’s calibrators and quality control material (c311, Roche Diagnostics Burgess Hill, United Kingdom). Coefficient of variation over 2 levels was ≤2.6% for ferritin, ≤6.0% for unsaturated iron-binding capacity, ≤8.5% for iron, and <5.7% for sTFR. Total iron-binding capacity was calculated as the sum of serum iron and serum unsaturated iron-binding capacity. Transferrin saturation (TSAT), expressed as a percentage, was calculated by dividing the serum iron concentration by the total iron-binding capacity. Plasma hepcidin and erythropoietin were measured by immunoassay (Ella Protein Simplex, Biotechne, Abingdon, United Kingdom) with the coefficient of variation of quality control samples over 2 levels ≤7.6% and ≤6.5%, respectively.

### Definition of Iron Deficiency

In keeping with clinical trials and the European Society of Cardiology 2021 guidelines on heart failure, we defined iron deficiency as a ferritin level <100 ng/mL (“absolute iron deficiency”) or a TSAT <20% and a ferritin level of 100 to 299 ng/mL (“functional iron deficiency”).^10^ We also used an alternative definition based on sex-specific sTFR concentrations (male >4.70 mg/L and female >4.59 mg/L) based on the assay-specific upper limit of the normal reference range. Anemia was defined at baseline using sex-specific baseline hemoglobin thresholds (male <130 g/L and female <120 g/L).

### Trial Outcomes

The primary outcome was the composite of worsening heart failure or cardiovascular death, whichever occurred first. An episode of worsening heart failure was either an urgent visit resulting in intravenous therapy for heart failure, not leading to hospital admission, or unplanned hospitalization for heart failure. The secondary outcomes were the composite of heart failure hospitalization or cardiovascular death; the total number of recurrent heart failure hospitalizations (including repeat admissions) and cardiovascular deaths; change from baseline to 8 months in the total symptom score of the Kansas City Cardiomyopathy Questionnaire Total Symptom Score using a scale from 0 to 100, with a higher score indicating fewer symptoms and a 5 or greater point change considered clinically meaningful; the incidence of a composite worsening renal function outcome (not included in the present analysis as there were few of these events); and death from any cause. All cardiovascular end points and deaths were adjudicated by an independent blinded committee.

In the present analyses, we compared the effect of dapagliflozin with placebo on the primary composite outcome, the individual components of cardiovascular death and worsening heart failure events, total heart failure hospitalizations and cardiovascular death, and death from any cause.

### Statistical Analysis

Baseline characteristics were compared between iron status groups by using the Kruskal-Wallis test for continuous variables and the χ^2^ test for categorical variables. The cumulative incidence of the primary end point by treatment assignment in the iron status subgroups was analyzed and plotted graphically using the Kaplan-Meier method. The effect of dapagliflozin compared with placebo on each outcome was calculated as hazard ratio and 95% CI derived from Cox proportional hazards models adjusted for a history of hospitalization for heart failure (not included in the model for all-cause mortality) and treatment assignment and stratified by baseline diabetes status, as prespecified in the statistical analysis plan for the main trial. The effect of dapagliflozin compared with placebo according to change in iron status between baseline and 12 months was examined using a landmark analysis from 12 months using the same model including patients who were alive at 12 months. For the iron deficiency yes/no subgroup analyses, an interaction test was performed to assess for any modification of treatment effect by iron status. The effect of dapagliflozin on the primary outcome according to the level of several iron metabolism biomarkers (analyzed as continuous variables) was examined using a fractional polynomial analysis using the Stata mfpi command.^24^ The relationship between iron status at baseline and subsequent outcomes was shown by hazard ratio and 95% CI derived from Cox proportional hazards models adjusted for a history of hospitalization for heart failure and treatment assignment. For the end point of all-cause mortality, no adjustment for a history of hospitalization for heart failure was performed. The effect of treatment on the composite end point of recurrent (first and total) heart failure hospitalizations and cardiovascular death (ie, total events) was examined using a semiparametric proportional rates model.^25^ Further analyses were performed with additional adjustment for age, sex, heart rate, systolic blood pressure, body mass index, ischemic cause, left ventricular ejection fraction, New York Heart Association class, NT-proBNP level (log-transformed), atrial fibrillation, and eGFR. This model was repeated with additional adjustment for high-sensitivity troponin T concentrations (log-transformed). The effects of dapagliflozin on biomarkers relating to iron metabolism between baseline and 12 months are presented as a ratio of geometric means derived from linear regression models including adjustment for log-transformed baseline values. Because hemoglobin was not measured routinely at the 12-month visit but was measured at the end-of-trial visit, the last recorded measurement was used to calculate the effect of dapagliflozin on hemoglobin using the same method as the iron biomarkers. Changes in hematocrit were analyzed using a mixed model for repeated measurements (adjusted for baseline values, visit, randomized treatment and interaction of treatment, and visit with a random intercept and slope per patient) and the between treatment group difference at 12 months after randomization are presented by subgroup as least square means difference and 95% CI. The slope of change in eGFR from day 14 to day 720 of follow-up according to randomized treatment was analyzed using a mixed model for repeated measurements with the same adjustment factors as described above. The effect of dapagliflozin on the development of iron deficiency during follow-up was examined with a logistic regression model with randomized treatment as the only independent variable. The proportion of patients with a clinically meaningful improvement (≥5 point increase) or deterioration (≥5 point decrease) in Kansas City Cardiomyopathy Questionnaire Total Symptom Score was compared between treatments using an odds ratio with a 95% CI calculated using methods previously described.^23^ All analyses were performed using Stata version 16 (College Station, TX) and SAS version 9.4 (SAS Institute). A *P* value <0.05 was considered statistically significant. No adjustment for multiple comparisons was made, and as such, all results should be considered exploratory.

## Results

Of the 4744 patients randomized in DAPA-HF, 3009 had ferritin and TSAT measurements available at baseline, and 1314 of these participants (43.7%) were iron deficient, as defined by ferritin and TSAT criteria. Of the 1314 patients with iron deficiency, 926 (70.5%) had absolute iron deficiency (ferritin <100 ng/mL), and 388 (29.5%) had functional iron deficiency (TSAT <20% and ferritin 100–300 ng/mL). Using the alternative definition based on sTFR, 412 of 2888 participants (14.3%) with a baseline sTFR measurement had iron deficiency. The distributions of baseline ferritin, TSAT, and sTFR by sex are shown in Figure S1. The proportion of patients with iron deficiency at baseline in the dapagliflozin and placebo groups was identical (666/1525 [43.7%] versus 648/1484 [43.7%]).

### Patient Characteristics According to Iron Status at Baseline

The baseline characteristics of patients according to their iron status are shown in Table 1. Compared with those who did not have iron deficiency, patients who had iron deficiency were more likely to be female (27.9% versus 17.6% male) but were not older and had a similar heart rate and systolic blood pressure compared with those without iron deficiency. People with iron deficiency were more likely to have an eGFR <60 mL·min·1.73 m^2^ (45.7% versus 36.7%, respectively) and to have anemia (35.5% versus 20.7%), coronary heart disease, hypertension, and diabetes. Patients with iron deficiency had worse New York Heart Association functional class (New York Heart Association class III/IV, 36.3% versus 26.3%) and markedly lower (worse) Kansas City Cardiomyopathy Questionnaire scores than those without, although previous heart failure hospitalization was not more common among those with iron deficiency. Baseline NT-proBNP level was higher in participants with iron deficiency, although atrial fibrillation was no more common and left ventricular ejection fraction was slightly higher than in patients without iron deficiency. High-sensitivity troponin T levels were higher in patients with iron deficiency compared with those who were iron replete at baseline. The prescription of key treatments was similar in the 2 groups, except for use of diuretics (more common) and mineralocorticoid receptor antagonists (less frequent) in individuals with iron deficiency. The use of anticoagulants did not differ between the 2 groups, but antiplatelet therapy was used more often in patients with iron deficiency. Proton pump inhibitor use was more common in patients with iron deficiency (36.5%) than in those without (28.7%). A greater number of patients with baseline iron deficiency, compared with those without, received treatments for iron deficiency or anemia during follow-up (including patients taking these at the time of randomization): intravenous iron in 16 (1.2%) versus 4 (0.2%), oral iron in 97 (7.4%) versus 67 (4.0%), vitamin B_12_ in 37 (2.8%) versus 31 (1.8%), and folic acid in 55 (4.2%) versus 49 (2.9%). The baseline characteristics for patients in whom iron deficiency was not determinable, compared with those with an assessment of iron status, are displayed in Table S1.

**Table 1.**
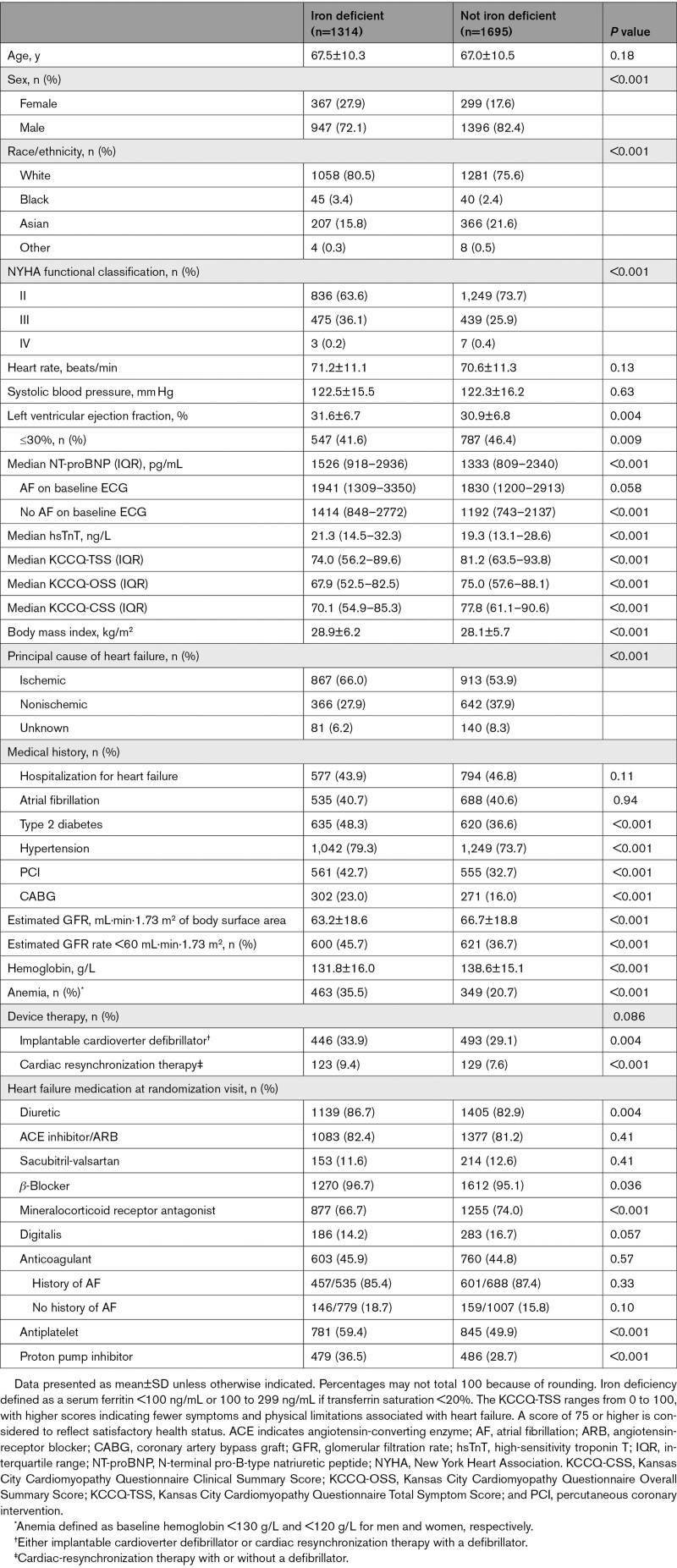
Baseline Characteristics According to the Presence of Iron Deficiency

### Cardiovascular Outcomes According to Iron Status at Baseline

**Figure 1.**
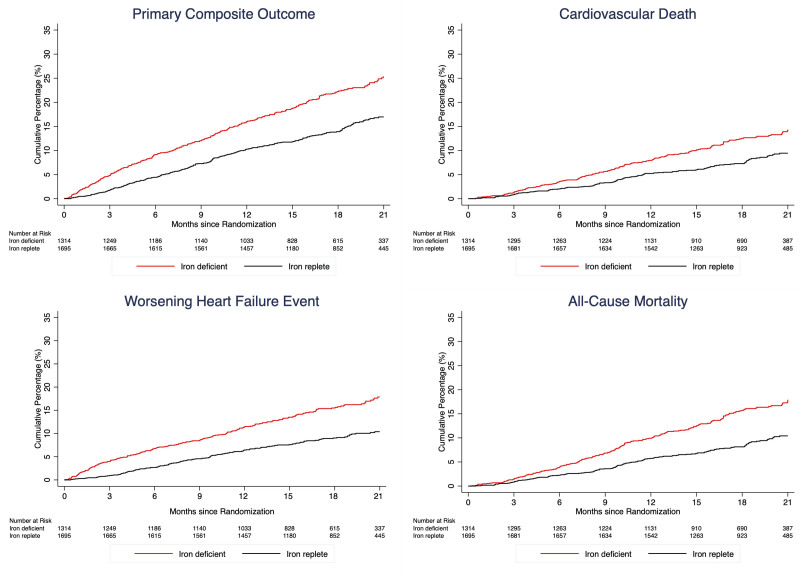
**Cardiovascular outcomes according to the presence of iron deficiency.** The primary outcome was a composite of death from cardiovascular causes, hospitalization for heart failure, or an urgent visit resulting in intravenous therapy for heart failure. The cumulative incidences of the primary outcome, worsening heart failure event, death from cardiovascular causes, and death from any cause were estimated with the use of the Kaplan-Meier method.

The cumulative incidence of the primary and secondary morbidity/mortality end points are shown in Figure 1 and Table 2. For each end point, the risk was higher in participants with iron deficiency compared with those without in both unadjusted and adjusted analyses. A sensitivity analysis using the alternative definition of iron deficiency based on sTFR showed similar findings (Table S2).

**Table 2.**
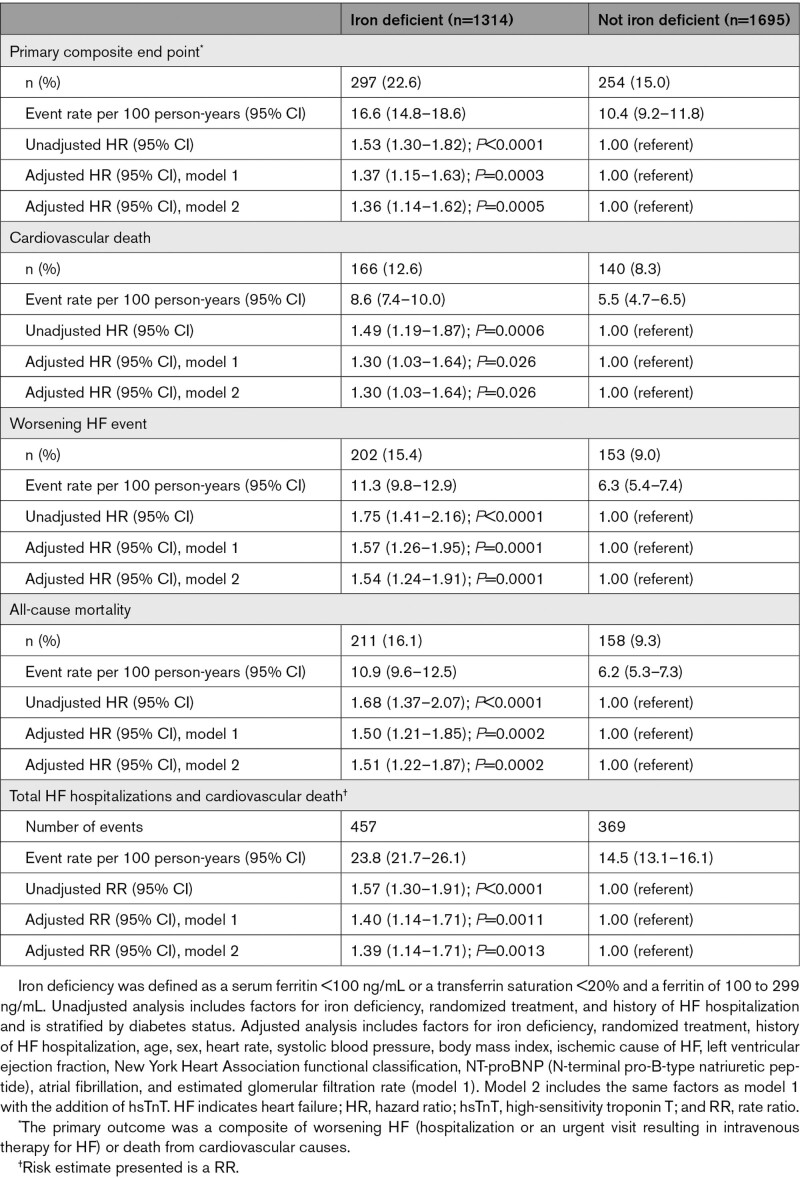
Cardiovascular Outcomes According to the Presence of Iron Deficiency

### eGFR Slopes According to Iron Status at Baseline

The rate of decline in kidney function over time was similar in patients with iron deficiency at baseline (–1.74 [95% CI, –2.16 to –1.32] mL·min·1.73 m^2^ per year) and in those without (–2.01 [95% CI, –2.38 to –1.66] mL/min/1.73 m^2^ per year; interaction *P*=0.32).

### Effects of Dapagliflozin on Cardiovascular Outcomes According to Baseline Iron Status

**Figure 2.**
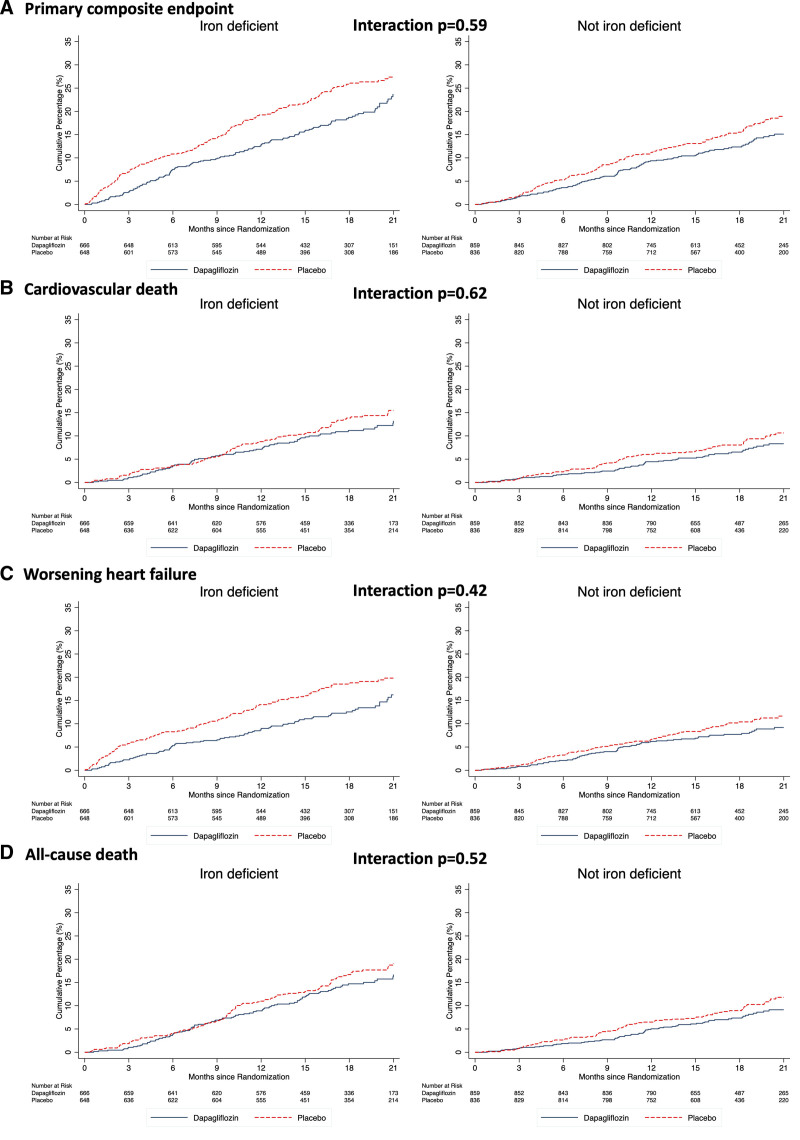
**Effect of dapagliflozin, compared with placebo, on cardiovascular outcomes according to the presence of iron deficiency. A**, Primary composite outcome. **B**, Cardiovascular death. **C**, Worsening heart failure event. **D**, All-cause death. The primary outcome was a composite of death from cardiovascular causes, hospitalization for heart failure, or an urgent visit resulting in intravenous therapy for heart failure. The cumulative incidences of the primary outcome, worsening heart failure event, death from cardiovascular causes, and death from any cause were estimated with the use of the Kaplan-Meier method. The interaction *P* value presented represents a treatment–by–iron deficiency interaction term.

The effects of dapagliflozin, compared with placebo, in patients with and without iron deficiency are shown in Figure 2 and Table 3.

**Table 3.**
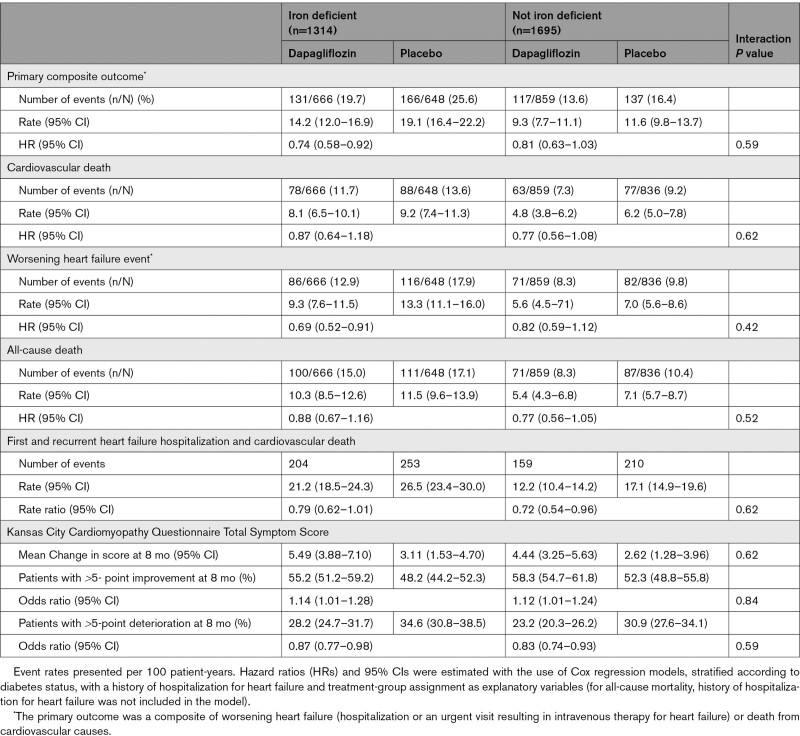
Effect of Dapagliflozin, Compared With Placebo, on Clinical Outcomes According to the Presence of Iron Deficiency at Baseline

**Figure 3.**
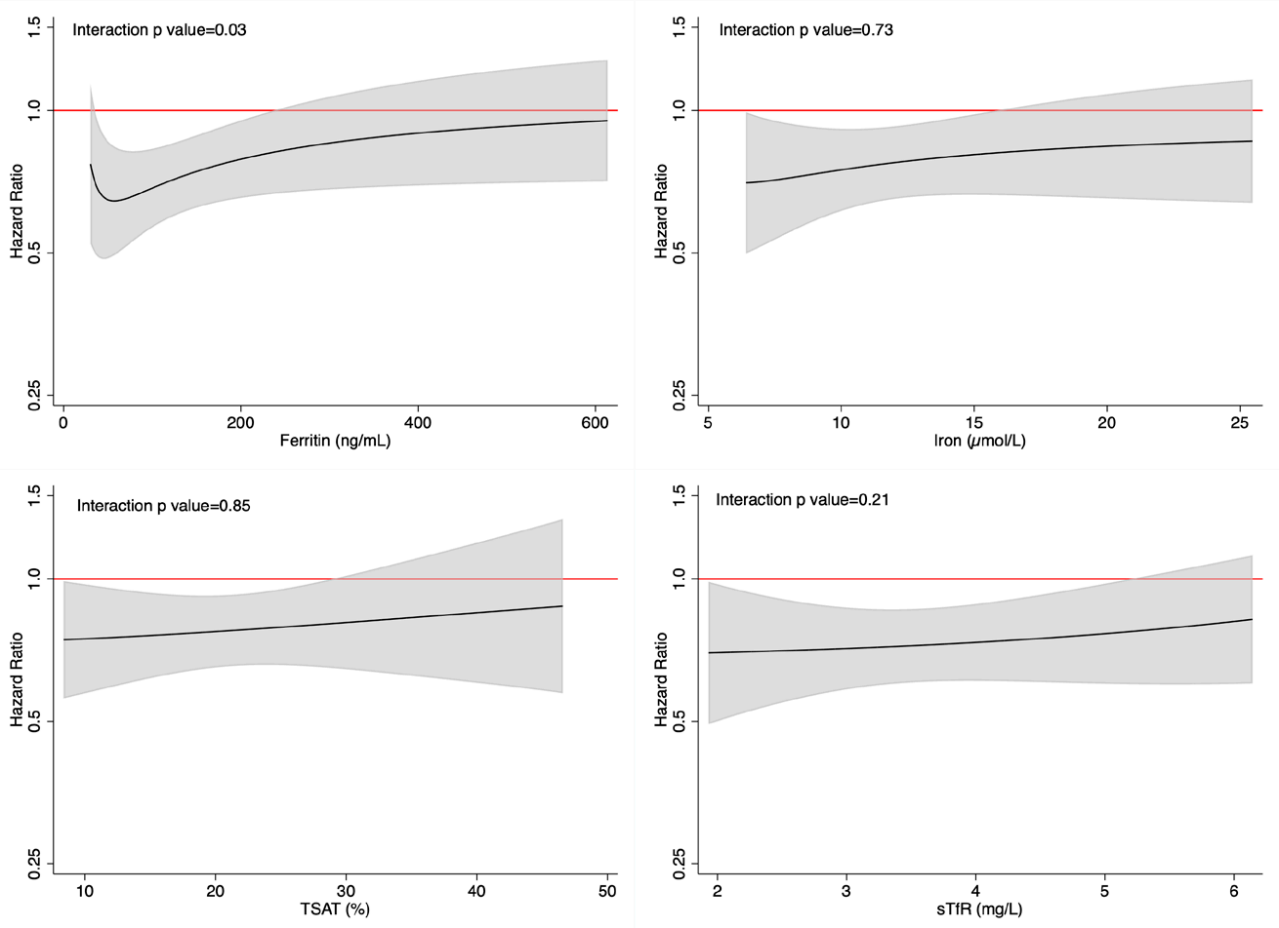
**Effect of dapagliflozin on the primary outcome according to baseline levels of iron metabolism biomarkers.** The solid black line represents a continuous hazard ratio, and the solid red line represents a hazard ratio of 1 (ie, no difference between treatments). The shaded area represents the 95% CI around the hazard ratio. The hazard ratio represents the treatment effect of dapagliflozin compared with placebo for the primary composite outcome.

The beneficial effect of dapagliflozin, compared with placebo, on the primary end point (hazard ratio, 0.74 [95% CI, 0.65–0.85] in the trial overall) was consistent in patients with (hazard ratio, 0.74 [95% CI, 0.58–0.92]) and without (hazard ratio, 0.81 [95% CI, 0.63–1.03]) iron deficiency (interaction *P*=0.59). A sensitivity analysis using the alternative definition of iron deficiency on the basis of sTFR gave similar findings (Table S3). The beneficial effect was also generally consistent across the range of ferritin, iron, TSAT, and sTFR levels at baseline when examined as a continuous variable (Figure 3), although there was a nominally significant interaction between baseline ferritin level and the effect of dapagliflozin, with a larger reduction in the primary outcome among patients with lower ferritin concentrations (Figure 3).

The effect of dapagliflozin on each of the components of the primary outcome and the secondary outcomes was generally similar for individuals with and without iron deficiency (Figure 2 and Table 3).

Because of the much higher event rate in patients with iron deficiency, the absolute risk reductions with dapagliflozin compared with placebo were larger than in iron-replete patients, ie, 5.0 versus 3.0 per 100 person-years for the primary composite outcome, 1.7 versus 1.1 per 100 person-years for cardiovascular death, and 4.0 versus 2.1 per 100 person-years for worsening heart failure.

### Effects of Dapagliflozin on Kansas City Cardiomyopathy Questionnaire Total Symptom Score According to Baseline Iron Status

Eight months after randomization, the mean increase (improvement) in Kansas City Cardiomyopathy Questionnaire Total Symptom Score with dapagliflozin compared with placebo, was +2.37 (95% CI, 0.11–4.63) in patients with iron deficiency and was similar to those without (+1.82 [95% CI, 0.03–3.61]; interaction *P*=0.62; Table 3).

### Effects of Dapagliflozin on Hematocrit, Hemoglobin, and Anemia According to Iron Status

**Figure 4.**
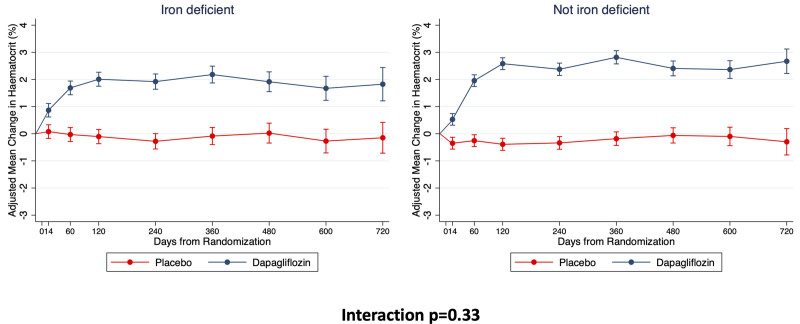
**Effect of dapagliflozin, compared with placebo, on hematocrit according to the presence of iron deficiency.** Means and 95% CIs were derived from a mixed-effect model adjusted for baseline values, visit, randomized treatment, and interaction of treatment and visit with a random intercept and slope per patient. Least-square mean changes along with 95% CI are shown.

The effects of dapagliflozin compared with placebo on hematocrit and markers of iron metabolism are shown in Figure 4 and Table 4. Between baseline and 12 months, there was a significant increase in hematocrit in the dapagliflozin group compared with the placebo group. The mean placebo-corrected increase in hematocrit at 12 months was 2.62% (95% CI, 2.40–2.85) and was not different between participants with iron deficiency (2.27% [95% CI, 1.82–2.71]) and those without iron deficiency (3.00% [95% CI, 2.65–3.35]; interaction *P*=0.10; Figure 4). The placebo-corrected increase in hemoglobin was 7.0 (95% CI, 6.2–7.9) g/L using the last available measurement during trial follow-up. This increase tended to be higher in iron-replete patients (7.34 [95% CI, 6.11–8.58] g/L) compared with those with iron deficiency at baseline (5.42 [95% CI, 3.77–7.08] g/L; interaction *P*=0.06). The effect of dapagliflozin on increasing hemoglobin was consistent whether patients had absolute or functional iron deficiency (interaction *P*=0.56). Using hematocrit-based definitions of anemia,^14^ dapagliflozin more frequently led to a reversal of anemia both in patients with iron deficiency at baseline (odds ratio, 2.43 [95% CI, 1.57–3.75]) and those iron-replete (odds ratio, 2.17 [95% CI, 1.40–3.37]; interaction *P*=0.72).

**Table 4.**
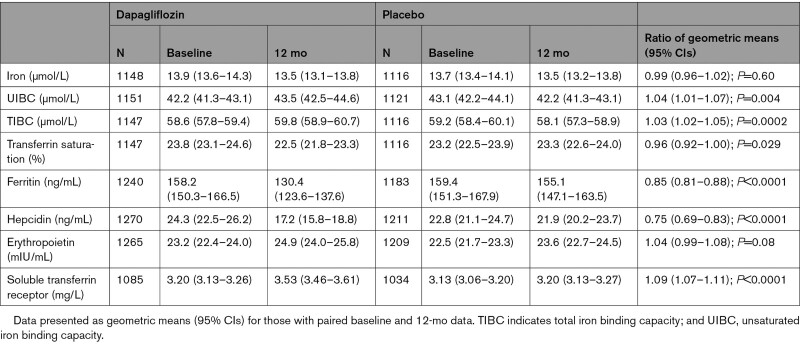
Effect on Dapagliflozin, Compared With Placebo, on Biomarkers Relating to Iron Metabolism

### Effects of Dapagliflozin on Markers of Iron Metabolism

There were reductions in TSAT, ferritin, and hepcidin, along with an increase in total iron-binding capacity and sTFR, in the dapagliflozin group compared with placebo, and a trend to an increase in erythropoietin (Table 4). There was no significant difference in serum iron concentrations between the groups. These changes were largely consistent in patients with and without iron deficiency, and with and without anemia, at baseline (Tables S4 and S5).

In those without iron deficiency at baseline (n=1334), 196 (28.4%) and 120 (18.6%) in the dapagliflozin and placebo groups, respectively, had new iron deficiency at 12 months defined using ferritin and TSAT criteria; the odds ratio (dapagliflozin versus placebo) for the development of iron deficiency by 12 months was 1.74 (95% CI, 1.34–2.25), *P*<0.001. The corresponding odds ratio using the sTFR-based definition was 2.26 (95% CI, 1.57–3.26), *P*<0.001.

### Outcomes Associated With the Development of Iron Depletion During Follow-Up

In a landmark analysis at 1 year after randomization, the rate of the primary outcome in patients without iron deficiency at baseline (defined using ferritin and TSAT criteria) who developed iron deficiency (n=315 [23.7%]), irrespective of treatment allocation, was higher than in those who remained iron-replete during follow-up (n=1016 [76.3%]): 17.0 (95% CI, 12.0–24.2) per 100 person-years versus 9.0 (95% CI, 6.9–11.8) per 100 person-years, giving an adjusted hazard ratio of this outcome for the development of iron deficiency versus not developing iron deficiency of 1.92 (95% CI, 1.21–3.04). Of the patients who had iron deficiency at baseline, the event rate in those who still had iron deficiency (n=716 [76.3%]) was 17.8 (95% CI, 14.2–22.2) per 100 person-years and 16.6 (95% CI, 11.0–24.9) per 100 person-years in those who had resolution of iron deficiency at 12 months (n=222 [23.7%]).

### Adverse Events According to Baseline Iron Status

Regardless of randomized treatment group, patients with iron deficiency were more likely to discontinue treatment overall, and to discontinue treatment because of an adverse event (Table S6). Prespecified adverse events of interest were generally similar for dapagliflozin and placebo, regardless of the presence of iron deficiency at baseline.

## Discussion

In this post hoc analysis of DAPA-HF, 43.7% of participants had iron deficiency at baseline, as defined using the ferritin and TSAT criteria recommended in contemporary heart failure guidelines.^10,11^ This prevalence is consistent with other recent reports using the same diagnostic criteria. For example, in a recent study from Sweden, iron deficiency was present in 46.3% of ambulatory patients with HFrEF.^26^ In a large Australian primary care study of patients with heart failure, the prevalence of iron deficiency, defined in the same way, was 39.7%, and among people with self-reported heart failure in NHANES (National Health and Nutrition Examination Survey 2017–2018), the prevalence of iron deficiency was 48.2% (95% CI, 36.8%–59.7%).^27,28^ A recent study from the United Kingdom reported an even higher prevalence, with 61% in patients with HFrEF iron-deficient.^29^ Conversely, using an alternative definition based on sTFR, we found a much lower prevalence of iron deficiency. However, there are few studies of sTFR levels in heart failure and variability between assays, resulting in uncertainty about the cut point to define iron deficiency using this approach.^30,31^

Much less is known about the incidence of new iron deficiency, which occurred in 18.6% of our patients assigned to placebo during 1 year, although the best criteria to diagnose true iron deficiency are under debate.^32^ Using a different definition of iron deficiency (serum iron ≤13 µmol/L), Graham and colleagues reported that 30.5% of patients with heart failure developed iron deficiency during 1 year.^33^ The proportion of patients in the placebo group in DAPA-HF with new iron deficiency at 12 months using this definition was 34.7%. Using a TSAT <20% to define iron deficiency, Fitzsimons and colleagues observed that 20% of patients developed incident iron deficiency during 6 months; the corresponding proportion in the present study using the same definition was 20.4% among patients assigned to placebo.^34,35^ These findings suggest that irrespective of definition, the short-term incidence, as well as prevalence, of iron deficiency is high in patients with heart failure.

Although iron deficiency is known to be associated with worse outcomes, the association with all-cause death, particularly after adjustment for other prognostic variables, has been an inconsistent finding, and recently there has been controversy about whether all biomarker definitions of iron deficiency carry the same prognostic import.^29,35–37^ Indeed, the predictive value of the combined ferritin and TSAT criteria used in this report has been challenged.^29,35–37^ However, we found iron deficiency, defined using ferritin and TSAT criteria, to be a statistically significant independent predictor of death (and other outcomes) even in models with extensive adjustment for other known prognostic variables including NT-proBNP and, we believe for the first time, high-sensitivity troponin T as well.

However, the most novel findings in the present study relate to the effect of dapagliflozin on indices of iron metabolism, given the evidence that agents of this type may stimulate erythropoiesis.^15–19,38^ Several studies have shown an early and transient rise in circulating erythropoietin levels after starting treatment with an SGLT2 inhibitor, and this seems to precede an increase in reticulocyte count and subsequently hematocrit and hemoglobin.^15–19,38^ If this is correct, SGLT2 inhibitor treatment should also increase the use of iron for erythropoiesis. Our findings are consistent with this. Although there was only a borderline significant increase in erythropoietin, other studies suggest that an earlier peak may have been missed because this measurement was only repeated 12 months after starting treatment in DAPA-HF.^16,17^ However, erythropoiesis, leading to increased mobilization and use of iron, would be expected to cause an increase in soluble transferrin receptor levels and iron-binding capacity, coupled with a decrease in ferritin, TSAT, and hepcidin, all of which were observed in DAPA-HF. Consistent with this hypothesis of greater use of iron after SGLT2 inhibition, treatment with dapagliflozin led to a higher incidence of iron deficiency during 12 months, defined using ferritin and TSAT criteria, compared with treatment with placebo. These observations raise the following question: What, if any, is the clinical significance of these biomarker changes, and our hypothesis to explain them? We believe there are 3 key considerations. First, does the increase in hematocrit/hemoglobin contribute to the benefit of SGLT2 inhibitors? Second, does iron deficiency as defined in this study attenuate the rise in hematocrit/hemoglobin in response to SGLT2 inhibition? Third, are the biomarker changes suggestive of iron deficiency induced by dapagliflozin clinically relevant? Although it has been suggested that the increase in hematocrit contributes to the benefits of SGLT2 inhibitors, this possibility is based on the results of mediation analysis and remains hypothetical.^39,40^ Although erythropoiesis may be restricted by iron deficiency, as recognized in the treatment of patients with chronic kidney disease, we did not find clear evidence of this in the patients in DAPA-HF.^20,21^ The mean placebo-corrected increase in hematocrit at 12 months did not differ significantly between iron-replete participants and those who were iron deficient (3.00% versus 2.27%; *P*-interaction=0.10), although there was a trend to a greater increase in hemoglobin (7.34 versus 5.42 g/L; *P*-interaction=0.06). Last, incident iron deficiency, as defined by changes in ferritin and TSAT, was associated with worse outcomes irrespective of treatment allocation. Although confounded, these findings are consistent with those of another recent study and with the randomized controlled trials using intravenous ferric carboxymaltose to treat iron deficiency.^34,36^ It is important to note, however, that the relative risk reductions in the primary and secondary morbidity and mortality end points with dapagliflozin, compared with placebo, were consistent in patients with and without iron deficiency at baseline (and the absolute risk reductions with dapagliflozin in individuals with iron deficiency were substantial, as a result of the high rates of death and hospitalization in these patients). Furthermore, the finding of a consistent benefit of dapagliflozin by iron status at baseline means that preexisting iron deficiency should not be considered as a barrier to the initiation of dapagliflozin or the development of iron deficiency during treatment as a reason to discontinue.

International guidelines already recommend testing for and treating iron deficiency in patients with HFrEF.^10,11^ Although none of our individual findings are definitive, collectively they do raise the question whether testing for and treating iron deficiency after the initiation of SGLT2 inhibition might also be desirable. As we have reported previously, dapagliflozin leads to the correction of anemia in many but not all patients with HFrEF, and the beneficial effect of dapagliflozin was consistent in those with and without anemia at baseline.^14^ In the present study, the effect of dapagliflozin on reversing anemia was consistent regardless of the presence or not of iron deficiency at baseline. Our new observations raise the potential for a therapeutic synergy between iron replenishment and SGLT2 inhibition in some patients with HFrEF not just to avoid iron deficiency but perhaps also to treat anemia.

### Limitations

As with all studies of this type, there are inherent limitations. Analysis of iron deficiency was not prespecified. Reticulocyte count, mean corpuscular hemoglobin concentration, and other indices that might have helped confirm an erythropoietic response to dapagliflozin were not measured in DAPA-HF. Ferritin levels can also reflect metabolic liver disease, which may be improved by SGLT2 inhibitors.^41,42^ We did not have serial measurements of iron indices and hemoglobin over time and may have missed earlier changes in some of these before the 1-year time point. Dapagliflozin improved survival compared with placebo, resulting in some survivor bias in our analyses.

### Conclusions

In conclusion, the prevalence and incidence of iron deficiency were high among patients with HFrEF in DAPA-HF, and participants with iron deficiency had worse outcomes than those who were iron-replete. Dapagliflozin appeared to increase iron use but improved outcomes, irrespective of iron status at baseline.

## Article Information

### Sources of Funding

The DAPA-HF trial was funded by AstraZeneca. Dr McMurray is supported by a British Heart Foundation Center of Research Excellence grant (RE/18/6/34217).

### Disclosures

Dr Docherty’s employer, the University of Glasgow, has been remunerated by AstraZeneca for working on the DAPA-HF trial. He has received speaker’s fees from AstraZeneca and has received grant support from Boehringer Ingelheim. Dr Verma has received research or speaking honoraria from Amgen, Amarin, AstraZeneca, Bayer, CMS, Janssen, HLS, Sanofi, Novo Nordisk, Novartis, Merck, and PhaseBio. He is also the president of the Canadian Medical and Surgical Knowledge Translation Research Group and holds the Tier 1 Canada Research Chair in Cardiovascular Surgery. Dr De Boer’s employer, the University Medical Center and University of Groningen, has received research grants or fees from AstraZeneca, Abbott, Boehringer Ingelheim, Cardior Pharmaceuticals GmbH, Ionis Pharmaceuticals, Inc, Novo Nordisk, and Roche (outside the submitted work). Dr de Boer received speaker’s fees from Abbott, AstraZeneca, Bayer, Novartis, and Roche (outside the submitted work). Dr O’Meara reports consulting and speaker’s fees from AstraZeneca, Bayer, Boehringer Ingelheim and Novartis during this trial, and participating as national lead investigator or member of the steering committee (fees paid to her institution in both cases) for the DAPA-HF trial (AstraZeneca) and the HEART-FID trial (American Regent), respectively. Dr Køber reports payments to his institution from AstraZeneca and personal fees from Novartis and Bristol Myers Squibb as a speaker outside the submitted work. Dr Kosiborod reports personal fees from AstraZeneca; grants, personal fees, and other fees from AstraZeneca; grants and personal fees from Boehringer Ingelheim; and personal fees from Sanofi, Amgen, Novo Nordisk, Merck (Diabetes), Janssen, Bayer, Novartis, Applied Therapeutics, Amarin, Eli Lilly, and Vifor Pharma outside the submitted work. Dr Martinez reports personal fees from AstraZeneca. Dr Ponikowski reports personal fees from AstraZeneca and clinical trial participation with AstraZeneca during the conduct of the study; clinical trial participation with and personal fees from Boehringer Ingelheim, Vifor Pharma, Bayer, RenalGuard, BMS, Cibiem, and Novartis; and personal fees from Respicardia, BERLIN-CHEMIE, Pfizer, and Servier outside the submitted work. Dr Sabatine reports research grant support through Brigham and Women’s Hospital from Amgen, Anthos Therapeutics, AstraZeneca, Bayer, Daiichi Sankyo, Eisai, Intarcia, Medicines Company, MedImmune, Merck, Novartis, Pfizer, Quark Pharmaceuticals, and Takeda, and consulting for Althera, Amgen, Anthos Therapeutics, AstraZeneca, Bristol Myers Squibb, CVS Caremark, DalCor, Dr. Reddy’s Laboratories, Dyrnamix, Esperion, IFM Therapeutics, Intarcia, Janssen Research and Development, Medicines Company, MedImmune, Merck, and Novartis. Dr Morrow reports grants to the TIMI Study Group from Abbott Laboratories, Amgen, Anthos Therapeutics, AstraZeneca, BRAHMS, Eisai, GlaxoSmithKline, Medicines Company, Merck, Novartis, Pfizer, Roche Diagnostics, Quark, Siemens, and Takeda, and consultant fees from In-Cardia, Merck & Co, Novartis, and Roche Diagnostics. Drs Sabatine and Morrow are members of the TIMI Study Group, which has received institutional research grant support through Brigham and Women’s Hospital from Abbott, Amgen, Anthos Therapeutics, ARCA Biopharma, AstraZeneca, Bayer, Daiichi-Sankyo, Eisai, Intarcia, Ionis Pharmaceuticals, MedImmune, Merck, Novartis, Pfizer, Quark Pharmaceuticals, Regeneron Pharmaceuticals, Roche, Siemens Healthcare Diagnostics, Medicines Company, and Zora Biosciences. Dr Schou reports personal fees and nonfinancial support from AstraZeneca and personal fees from Novo Nordisk and Boehringer Ingelheim outside the submitted work. Drs Bengtsson, Hammarstedt, Langkilde, Lindholm, Little, and Sjöstrand are full-time employees of AstraZeneca. Dr Solomon reports grants from AstraZeneca during the conduct of the study, grants and personal fees from Alnylam, Amgen, AstraZeneca, BMS, Gilead, GSK, MyoKardia, Novartis, Theracos, Bayer, and Cytokinetics; grants from Bellerophon, Celladon, Ionis, Lone Star Heart, Mesoblast, the National Heart, Lung, and Blood Institute/National Institutes of Health, and Sanofi Pasteur Eidos; and personal fees from Akros, Corvia, Ironwood, Merck, Roche, Takeda, Quantum Genomics, AoBiome, Janssen, Cardiac Dimensions, Tenaya, Daichi Sankyo, Cardurion, and Eko.Ai outside the submitted work. Dr Sattar has consulted for Amgen, AstraZeneca, Boehringer Ingelheim, Eli Lilly, Merck Sharp and Dohme, Novo Nordisk, Novartis, Sanofi, and Pfizer; and has received grant support from Boehringer Ingelheim. Dr Jhund’s employer, the University of Glasgow, has been remunerated by AstraZeneca for working on the DAPA-HF and DELIVER trials. He has received personal fees from Novartis and Cytokinetics and grants from Boehringer Ingelheim outside the submitted work. Dr McMurray reports payments to his employer, Glasgow University, for work on clinical trials, consulting, lecturing and other activities: Alnylam, Amgen, AstraZeneca, Bayer, Boehringer Ingelheim, BMS, Cardurion, Cytokinetics, Dal-Cor, GSK, Ionis, KBP Biosciences, Novartis, Pfizer, and Theracos. He has received personal lecture fees from Abbott, Hikma, Sun Pharmaceuticals, and Servier. The other author reports no conflicts.

### Supplemental Material

Tables S1–S6

Figure S1

## Supplementary Material


