# Supplementary material for: Iron Deficiency in Heart Failure and Effect of Dapagliflozin: Findings From DAPA-HF
Source: Circulation. 2022 Aug 16;146(13):980–94. doi: 10.1161/CIRCULATIONAHA.122.060511 (PMC9508991; doi:10.1161/CIRCULATIONAHA.122.060511)

Iron deficiency in heart failure and effect of dapagliflozin: Findings from DAPA-HF

SUPPLEMENTARY MATERIAL

Supplement Table 1: Baseline characteristics according to availability of iron status

	Iron status not available (n=1735)	Iron status available (n=3009)	P value
Age – yr	64.8±11.4	67.2±10.4	<0.001
Sex – no (%)			0.008
Female	443 (25.5)	666 (22.1)	
Male	1,292 (74.5)	2,343 (77.9)	
Race – no (%)			<0.001
White	994 (57.3)	2,339 (77.7)	
Black	141 (8.1)	85 (2.8)	
Asian	543 (31.3)	573 (19.0)	
Other	57 (3.3)	12 (0.4)	
NYHA functional classification – no. (%)			<0.001
II	1,118 (64.4)	2,085 (69.3)	
III	584 (33.7)	914 (30.4)	
IV	33 (1.9)	10 (0.3)	
Heart rate – beats/min	72.6±12.4	70.9±11.2	<0.001
Systolic Blood Pressure – mmHg	120.8±17.1	122.4±15.9	0.002
Left ventricular ejection fraction – %	30.8±6.8	31.2±6.8	0.060
≤30% – no. (%)	827 (47.7)	1,334 (44.3)	0.026
Median NT-proBNP (IQR) – pg/ml	1486 (868-2802)	1419 (850-2567)	0.075
AF on baseline ECG	2070 (1298-3229)	1884 (1230-3147)	0.17
No AF on baseline ECG	1314 (765-2546)	1276 (774-2350)	0.18
Median KCCQ-TSS (IQR)	77.1 (56.2-91.7)	78.1 (60.4-91.7)	0.001
Median KCCQ-OSS (IQR)	68.3 (50.4-83.3)	72.1 (55.0-86.2)	<0.001
Median KCCQ-CSS (IQR)	73.0 (54.2-87.5)	75.0 (58.3-88.9)	0.001
Body-mass index – kg/m^2	27.6±6.0	28.5±5.9	<0.001
Principal cause of heart failure – no. (%)			<0.001
Ischemic	894 (51.5)	1,780 (59.2)	
Non-ischemic	679 (39.1)	1,008 (33.5)	
Unknown	162 (9.3)	221 (7.3)	
Medical history – no. (%)			
Hospitalization for heart failure	880 (50.7)	1,371 (45.6)	<0.001
Atrial fibrillation	595 (34.3)	1,223 (40.6)	<0.001
Type 2 diabetes	728 (42.0)	1,255 (41.7)	0.87
Hypertension	1,232 (71.0)	2,291 (76.1)	<0.001
PCI	508 (29.3)	1,116 (37.1)	<0.001
CABG	226 (13.0)	573 (19.0)	<0.001
Estimated GFR – ml/min/1.73 m^2 of body-surface area	66.9±20.4	65.1±18.8	0.003
Estimated GFR rate < 60 ml/min/1.73 m^2 – no. (%)	705 (40.6)	1,221 (40.6)	0.98
Hemoglobin	135.3±16.7	135.6±15.9	0.53
Anemia – no. (%)*	490 (28.5)	812 (27.2)	0.34
Device therapy – no (%)			
Implantable cardioverter-defibrillator†	303 (17.5)	939 (31.2)	<0.001
Cardiac-resynchronization therapy‡	102 (5.9)	252 (8.4)	0.002
Heart failure medication at randomization visit – no (%)			
Diuretic	1,464 (84.4)	2,544 (84.5)	0.88
ACE-inhibitor/ARB	1,492 (86.0)	2,460 (81.8)	<0.001
Sacubitril-valsartan	141 (8.1)	367 (12.2)	<0.001
Beta-blocker	1,676 (96.6)	2,882 (95.8)	0.16
Mineralocorticoid receptor antagonist	1,238 (71.4)	2,132 (70.9)	0.71
Digitalis	418 (24.1)	469 (15.6)	<0.001
Anticoagulant – no (%)	606 (34.9)	1,363 (45.3)	<0.001

History of AF	471 (79.2)	1058 (86.5)	<0.001
No history of AF	135 (11.8)	305 (17.1)	<0.001
Antiplatelet – no (%)	966 (55.7)	1,626 (54.0)	0.27
Proton pump inhibitor	437 (25.2)	965 (32.1)	<0.001

Data presented as mean (SD) unless otherwise indicated.

Percentages may not total 100 because of rounding.

Iron deficiency defined as a serum ferritin <100 ng/mL or 100-299 ng/mL if transferrin saturation <20%

ACE = angiotensin-converting enzyme; ARB = angiotensin-receptor blocker; ARNI = angiotensin receptor-neprilysin inhibitor; CABG = coronary artery bypass graft; GFR = glomerular filtration rate; IQR = interquartile range; LVEF = left ventricular ejection fraction; MRA = mineralocorticoid receptor antagonist; NT-proBNP = N-terminal pro-B-type natriuretic peptide; NYHA = New York Heart association. KCCQ-TSS = Kansas City Cardiomyopathy Questionnaire total symptom score - range from 0 to 100, with higher scores indicating fewer symptoms and physical limitations associated with heart failure. A score of 75 or above is considered to reflect satisfactory health status; KCCQ-CSS = Kansas City Cardiomyopathy Questionnaire clinical summary score; KCCQ-OSS = Kansas City Cardiomyopathy Questionnaire overall summary score; PCI = percutaneous coronary intervention

* Anemia defined as baseline hemoglobin <130 g/L and <120 g/L for men and women , respectively
† Either implantable cardioverter-defibrillator or cardiac resynchronization therapy with a defibrillator.
‡ Cardiac-resynchronization therapy with or without a defibrillator

Supplement Table 2: Cardiovascular outcomes according to the presence of iron deficiency defined using sTFR

	Iron deficient (n=412)	Not iron deficient (n=2476)
**Primary composite endpoint***		
Event rate per 100 person-years	25.1 (21.1-29.8)	11.3 (10.2-12.4)
Unadjusted HR (95% CI)	2.10 (1.73-2.57)	1.00 (referent)
Adjusted HR (95% CI)	1.52 (1.23-1.87)	1.00 (referent)
**Cardiovascular death**		
Event rate per 100 person-years	12.5 (9.9-15.7)	5.9 (5.1-6.7)
Unadjusted HR (95% CI)	2.01 (1.54-2.62)	1.00 (referent)
Adjusted HR (95% CI)	1.43 (1.08-1.89)	1.00 (referent)
**Worsening HF event**		
Event rate per 100 person-years	17.6 (14.4-21.6)	7.1 (6.3-8.1)
Unadjusted HR (95% CI)	2.33 (1.83-2.97)	1.00 (referent)
Adjusted HR (95% CI)	1.65 (1.28-2.12)	1.00 (referent)
**All-cause mortality**		
Event rate per 100 person-years	15.1 (12.2-18.6)	7.1 (6.3-8.1)
Unadjusted HR (95% CI)	2.00 (1.57-2.54)	1.00 (referent)
Adjusted HR (95% CI)	1.49 (1.16-1.92)	1.00 (referent)
**Total heart failure hospitalizations and cardiovascular death†**		
Event rate per 100 person-years	34.7 (30.3-39.9)	15.9 (14.7-17.3)
Unadjusted RR (95% CI)	2.05 (1.64-2.56)	1.00 (referent)
Adjusted RR (95% CI)	1.48 (1.18-1.86)	1.00 (referent)

* The primary outcome was a composite of worsening heart failure (hospitalization or an urgent visit resulting in intravenous therapy for heart failure) or death from cardiovascular causes.

† Risk estimate presented is a rate ratio

Unadjusted analysis includes factors for iron deficiency, randomized treatment and history of HF hospitalization and is stratified by diabetes status

Adjusted analysis includes factors for iron deficiency, randomized treatment, history of HF hospitalization, age, sex, heart rate, systolic blood pressure, body mass index, ischemic etiology of heart failure, left ventricular ejection fraction, NYHA functional classification, NT-proBNP, atrial fibrillation, and estimated glomerular filtration rate.

Supplement Table 3: Effect of dapagliflozin, compared to placebo, on clinical outcomes according to the presence of iron deficiency defined using sTFR

	Iron deficient (n=412)		Not iron deficient (n=2476)		Interaction p value
	**Dapagliflozin**	**Placebo**	**Dapagliflozin**	**Placebo**	
**Primary composite outcome***					
Number of events (n/N)	60/210 (28.6)	71/202 (35.1)	177/1251 (14.1)	222/1225 (18.1)	
Rate (95% CI)	22.3 (17.3-28.8)	28.0 (22.2-35.3)	9.7 (8.4-11.2)	13.0 (11.4-14.8)	
HR (95% CI)	0.80 (0.56-1.12)		0.75 (0.62-0.91)		0.78
**Cardiovascular death**					
Number of events (n/N)	34/210 (16.2)	39/202 (19.3)	99/1251 (7.9)	119/1225 (9.7)	
Rate (95% CI)	11.5 (8.2-16.2)	13.5 (9.8-18.4)	5.2 (4.3-6.4)	6.5 (5.5-7.8)	
HR (95% CI)	0.87 (0.55-1.38)		0.79 (0.61-1.04)		0.74
**Worsening heart failure event***					
Number of events (n/N)	40/210 (19.0)	52/202 (25.7)	112/1251 (9.0)	140/1225 (11.4)	
Rate (95% CI)	14.9 (10.9-20.3)	20.5 (15.6-26.9)	6.1 (5.1-7.4)	8.2 (6.9-9.6)	
HR (95% CI)	0.72 (0.47-1.08)		0.75 (0.59-0.97)		0.82
**All-cause death**					
Number of events (n/N)	40/210 (19.0)	48/202 (23.8)	122/1251 (9.8)	143/1225 (11.7)	
Rate (95% CI)	10.3 (8.5-12.6)	11.5 (9.6-13.9)	5.4 (4.3-6.8)	7.1 (5.7-8.7)	
HR (95% CI)	0.88 (0.67-1.16)		0.77 (0.56-1.05)		0.52
**First and recurrent heart failure hospitalization and cardiovascular death**					
Number of events	89	113	256	333	

Rate (95% CI)	30.4 (24.7-37.4)	39.2 (32.6-47.1)	13.6 (12.0-15.3)	18.4 (16.5-20.5)	
Rate ratio (95% CI)	0.78 (0.53-1.13)		0.74 (0.59-0.92)		0.82
**KCCQ Total Symptom Score (TSS)**					
Mean Change in score at 8 months (95% CI)	7.14 (4.22-10.5)	3.82 (0.87-6.78)	4.55 (3.51-5.59)	2.67 (1.56-3.78)	0.32
Patients with ≥5, point improvement at 8 months (%)	55.7 (48.7-62.8)	49.2 (41.9-56.4)	57.1 (54.2-60.1)	50.9 (48.0-53.9)	
Odds ratio (95% CI)	1.13 (0.92-1.39)		1.13 (1.04-1.23)		0.99
Patients with ≥5-point deterioration at 8 months (%)	30.6 (24.0-37.1)	35.7 (28.8-42.6)	24.5 (22.0-26.9)	31.7 (29.0-34.4)	
Odds ratio (95% CI)	0.90 (0.72-1.11)		0.84 (0.76-0.92)		0.55

*The primary outcome was a composite of worsening heart failure (hospitalization or an urgent visit resulting in intravenous therapy for heart failure) or death from cardiovascular causes.

Event rates presented per 100 patient-years.

Supplement Table 4: Effect of dapagliflozin, compared with placebo, on biomarkers relating to iron metabolism by the presence or not of iron deficiency

	Iron deficient	Not iron deficient	
	**Ratio of geometric means (95%)**	**Ratio of geometric means (95%)**	**Interaction p value**
**Iron (umol/L)**	0.99 (0.94-1.05)	0.99 (0.95-1.03)	0.99
**UIBC (umol/L)**	1.02 (0.98-1.07)	1.05 (1.01-1.09)	0.48
**TIBC (umol/L)**	1.01 (0.99-1.04)	1.04 (1.02-1.07)	0.21
**Transferrin saturation (%)**	0.98 (0.92-1.04)	0.95 (0.91-1.00)	0.54
**Ferritin (ng/mL)**	0.85 (0.78-0.92)	0.84 (0.80-0.89)	0.87
**Hepcidin (ng/mL)**	0.69 (0.58-0.83)	0.77 (0.70-0.85)	0.25
**Erythropoietin (mIU/mL)**	1.05 (0.97-1.13)	1.03 (0.98-1.08)	0.61
**Soluble transferrin receptor (mg/L)**	1.09 (1.05-1.13)	1.09 (1.07-1.12)	0.91

Iron deficiency was defined as a serum ferritin < 100ng/mL or a TSAT <20% and a ferritin of 100-299 ng/mL.

Supplement Table 5: Effect of dapagliflozin, compared with placebo, on biomarkers relating to iron metabolism by the presence or not of anemia

	Anemia	No anemia	
	Ratio of geometric means (95%)	Ratio of geometric means (95%)	Interaction p value
**Iron (umol/L)**	0.97 (0.91-1.04)	1.00 (0.96-1.03)	0.65
**UIBC (umol/L)**	1.06 (1.01-1.12)	1.03 (1.00-1.07)	0.42
**TIBC (umol/L)**	1.05 (1.01-1.09)	1.03 (1.01-1.05)	0.41
**Transferrin saturation (%)**	0.93 (0.85-1.01)	0.97 (0.93-1.01)	0.41
**Ferritin (ng/mL)**	0.84 (0.76-0.92)	0.85 (0.81-0.89)	0.84
**Hepcidin (ng/mL)**	0.73 (0.60-0.89)	0.77 (0.69-0.85)	0.69
**Erythropoietin (mIU/mL)**	1.03 (0.93-1.13)	1.04 (0.99-1.09)	0.78
**Soluble transferrin receptor (mg/L)**	1.10 (1.04-1.15)	1.09 (1.06-1.11)	0.81

Supplement Table 6: Occurrence of study drug discontinuation and pre-specified adverse events by treatment group in patients by iron status at baseline

	Iron deficient		Not iron deficient		Interaction p value
	**Dapagliflozin (n=665)**	**Placebo (n=648)**	**Dapagliflozin (n=859)**	**Placebo (n=835)**	
Discontinuation of trial treatment for any reason	96 (14.4)	95 (14.7)	73 (8.5)	66 (7.9)	0.68
Discontinuation of trial treatment due to adverse event	41 (6.2)	51 (7.9)	33 (3.8)	23 (2.8)	0.08
Volume depletion	48 (7.2)	39 (6.0)	61 (7.1)	64 (7.7)	0.34
Renal adverse event	41 (6.2)	51 (7.9)	44 (5.1)	43 (5.1)	0.41
Fracture	14 (2.1)	19 (2.9)	22 (2.6)	18 (2.2)	0.28
Amputation	5 (0.8)	5 (0.8)	5 (0.6)	2 (0.2)	0.38
Major hypoglycaemia	0 (0.0)	1 (0.2)	1 (0.1)	1 (0.1)	-
Diabetic ketoacidosis	1 (0.2)	0 (0.0)	1 (0.1)	0 (0.0)	-

The safety population included all the patients who had undergone randomization and received at least one dose of dapagliflozin or placebo.

Supplement Figure 1: Distribution of iron biomarkers by sex

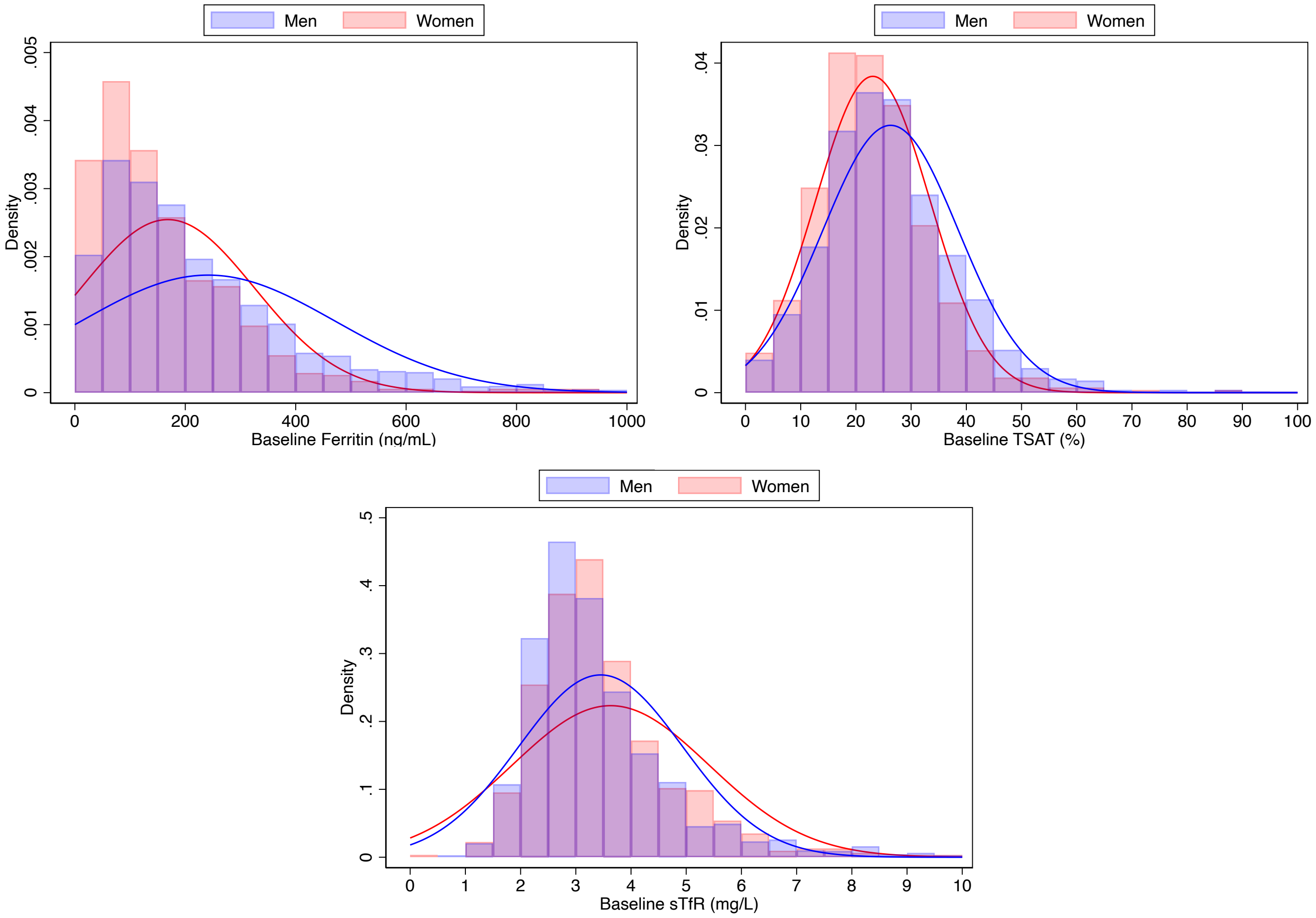

Supplement: Supplementary file 1 [file cir-146-980-s001.pdf]
